# Treatment of ruptured rectal artery aneurysm in a patient with neurofibromatosis

**DOI:** 10.1186/s42155-022-00317-y

**Published:** 2022-08-04

**Authors:** Hidehiko Nemoto, Kensaku Mori, Yohei Takei, Shunsuke Kikuchi, Sodai Hoshiai, Yoshiyuki Yamamoto, Takahito Nakajima

**Affiliations:** 1 Department of Radiology, University of Tsukuba Hospital, 2-1-1 Amakubo, Tsukuba, 305-8576 Japan; 2 Department of Radiology, Faculty of Medicine, University of Tsukuba, 1-1-1 Tennodai, Tsukuba, 305-8575 Japan; 3 Department of Radiology, Tsuchiura Kyodo General Hospital, 4-1-1 Ohtsuno, Tsuchiura, 300-0028 Japan; 4 Department of Gastroenterology, Faculty of Medicine, University of Tsukuba, 1-1-1 Tennodai, Tsukuba, 305-8575 Japan

**Keywords:** Superior rectal artery, Aneurysm, Rupture, Neurofibromatosis type 1, Embolization, Coils

## Abstract

**Background:**

Superior rectal artery (SRA) aneurysms are rare. Although melena is the most common symptom, it has not been observed in cases of aneurysms located in the SRA trunk. Here, we report a case of a ruptured SRA trunk aneurysm successfully treated with coil embolization. Including our case, three of the four reported cases of SRA trunk aneurysms were related to neurofibromatosis type 1 (NF1).

**Case presentation:**

A 52-year-old woman with NF1 was referred to our hospital for the investigation of an abdominal mass with back pain. She had previously undergone a blood transfusion at another hospital for anemia without melena. Computed tomography angiography revealed a ruptured SRA trunk aneurysm measuring 3 cm in diameter and surrounded by a retroperitoneal hematoma. The aneurysm was isolated by embolizing the SRA trunk distally and proximally. Distal embolization was performed retrogradely from the internal iliac artery (IIA) via the middle rectal artery (MRA)-SRA anastomosis because the antegrade approach from the inferior mesenteric artery (IMA) failed. To our knowledge, this is the first case of successful coil embolization of an IMA branch through the IIA.

**Conclusion:**

SRA trunk aneurysms are rare; however, they are frequently associated with NF1. Antegrade distal embolization beyond the aneurysm is sometimes difficult to achieve. In such cases, a retrograde approach via MRA-SRA anastomosis can be the choice for isolating SRA trunk aneurysms.

## Background

Neurofibromatosis type 1 (NF1) is an autosomal dominant multisystem disorder characterized by multiple café-au-lait macules, intertriginous freckling, and cutaneous neurofibromas. Less common but potentially more serious manifestations include optic nerve and other central nervous system gliomas; malignant peripheral nerve sheath tumors; scoliosis; tibial dysplasia; and gastrointestinal, endocrine, or pulmonary disease (Friedman JM et al. 1998). Vasculopathy, including aneurysm formation, also occurs in medium to large arteries. According to a review on aneurysms caused by neurofibromatosis (Bargiela et al. 2018), NF1-related aneurysms were observed in the head and neck in 27 of 58 patients, subclavian and intercostal arteries in 14 patients, visceral arteries in 8 patients, and other uncommon regions in 9 patients. In addition, to the best of our knowledge, a superior rectal artery (SRA) trunk aneurysm related to NF1 has been reported in two patients (Makino et al. 2013; Yow et al. 2015). Herein, we report the third case of a ruptured SRA trunk aneurysm related to NF1, emphasizing the importance of isolating aneurysms to avoid re-bleeding after coil embolization.

## Case presentation

**Fig. 1:**
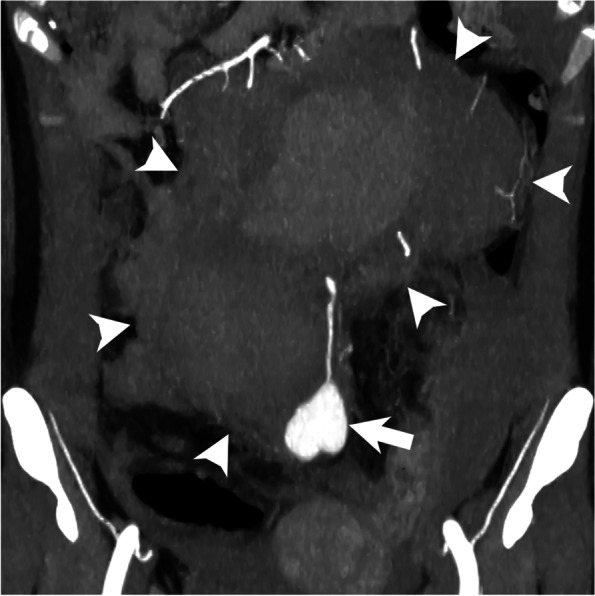
Coronal partial maximum intensity projection image reconstructed from computed tomography angiography showing a ruptured superior rectal artery trunk aneurysm (arrow) and a large retroperitoneal hematoma (arrowheads)

A 52-year-old woman with NF1 was referred to our hospital for an abdominal mass with back pain and anemia. She had undergone mastectomy for left breast cancer and colectomy for colon cancer, 3 and 17 years ago, respectively. In the previous hospital, she had hypotension (blood pressure = ~70 mmHg) with loss of consciousness, and unenhanced computed tomography revealed a retroperitoneal mass suspected to be a neurogenic tumor related to NF1. At that time, her hemoglobin level was almost normal at 11.6 g/dL. However, it gradually decreased to 6.1 g/dL over the next 6 days. She had received a total blood transfusion of 16 packed red blood cell units before admission to our hospital. On admission, her blood pressure and heart rate were 119/96 mmHg and 96 beats per minute, respectively. Laboratory data showed a hemoglobin level of 10.0 g/dL, platelet count of 629,000/μL, prothrombin time of 13.9 s, and activated partial thromboplastin time of 48.8 s. Computed tomography angiography (CTA) revealed a saccular SRA trunk aneurysm that measured 3 cm in diameter and was surrounded by an increased large retroperitoneal hematoma (Fig. 1). In addition, two hypodense masses protruding from the left second and third sacral foramen were observed, which were indicative of neurofibromas related to NF1 (not shown). Therefore, we treated the ruptured SRA trunk aneurysm with transcatheter arterial embolization (TAE).

**Fig. 2:**
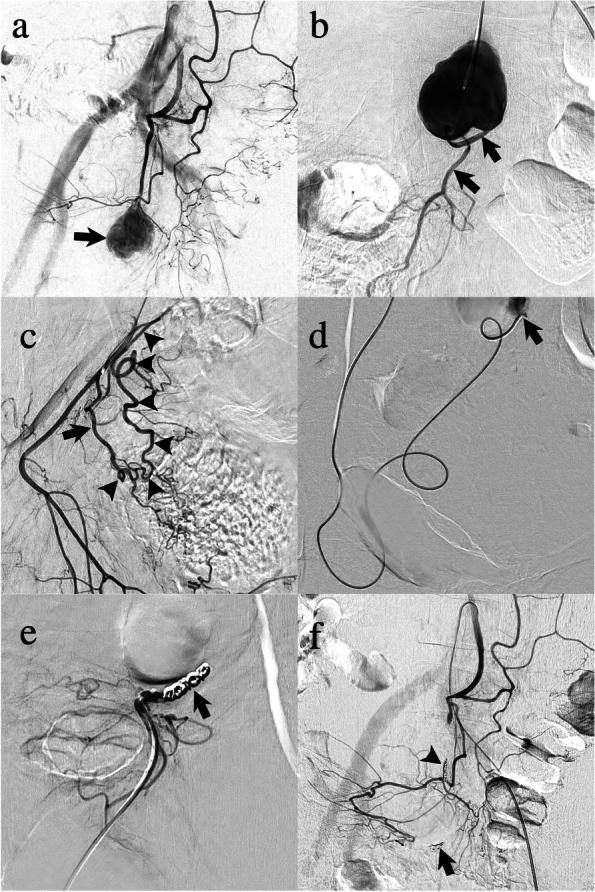
**a** Inferior mesenteric arteriogram showing the superior rectal artery (SRA) trunk aneurysm (arrow). **b** Aneurysmogram showing the distal SRA trunk (arrows). **c** Internal pudendal arteriogram showing the middle rectal artery (MRA) (arrow) and MRA-SRA anastomosis (arrowheads). **d** A high-flow type microcatheter is advanced into the SRA trunk distal to the aneurysm (arrow) via MRA-SRA anastomosis using the coaxial microcatheter system. **e** Retrograde arteriogram after coil embolization (arrow) showing complete occlusion of distal SRA. **f** The final inferior mesenteric arteriogram after proximal coil embolization showing complete cessation of blood flow into the aneurysm. Note that the coils are deployed distally (arrow) and proximally to the aneurysm (arrowhead)

**Fig. 3:**
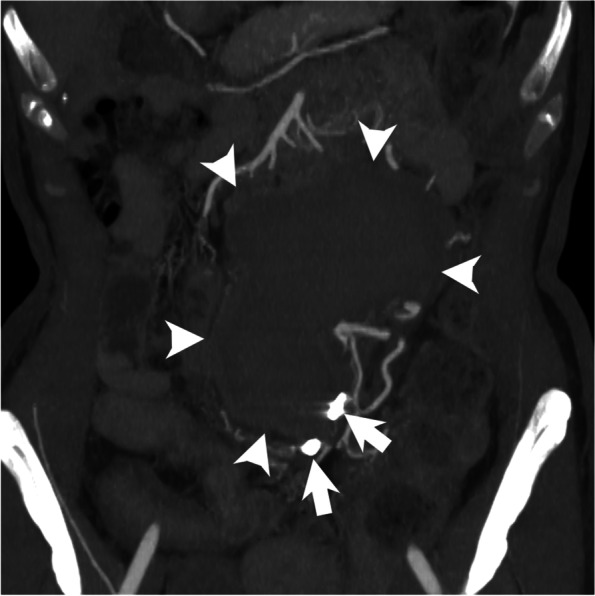
Coronal partial maximum intensity projection image reconstructed from computed tomography angiography obtained 3 months after transcatheter arterial embolization showing the lack of enhancement of the superior rectal artery trunk aneurysm. Note the coils deployed proximally and distally to the aneurysm (arrows) and decreased size of the retroperitoneal hematoma (arrowheads)

Under local anesthesia, employing the femoral artery approach, inferior mesenteric artery (IMA) angiography was performed using a 4-F shepherd hook-type catheter (Medikit Co. Ltd., Tokyo, Japan), and an SRA trunk aneurysm was confirmed (Fig. 2a). A 2.6-F high-flow-type microcatheter (Masters HF; ASAHI INTECC Co. Ltd., Aichi, Japan) was selectively advanced into the SRA over a 0.016-in micro guidewire (Meister; ASAHI INTECC Co. Ltd., Aichi, Japan). SRA angiography revealed the distal vessel of the aneurysm (Fig. 2b). To isolate the aneurysm, we attempted to embolize the distal vessel; however, microcatheter insertion failed via the antegrade approach. Meanwhile, the distal vessel disappeared on the aneurysmogram; this might have resulted from thrombogenesis or retrograde blood flow. Proximal embolization without isolation of the aneurysm was considered insufficient for hemostasis because re-bleeding due to retrograde blood flow could occur. Therefore, we attempted a retrograde approach to the distal vessel. A shepherd hook-type catheter was advanced into the right common iliac artery. Right internal iliac arteriography with a high-flow type microcatheter revealed the middle rectal artery (MRA) arising from the internal pudendal artery. The inferior pudendal arteriogram revealed anastomosis between the MRA and SRA (Fig. 2c). To pass through the narrow and tortuous anastomosis, a 1.6-F microcatheter (Marvel S; Tokai Medical Products Inc., Aichi, Japan) with a 0.014-in micro guidewire was inserted coaxially into the high-flow microcatheter. Using this double coaxial microcatheter system (Hongo et al. 2014), the high-flow microcatheter was advanced using the MRA-SRA anastomosis into the distal SRA trunk to be embolized with a coil (Interlock, 3 mm/12 cm, Boston Scientific, MA, USA) (Fig. 2d). Finally, the aneurysm was isolated with proximal embolization of the SRA trunk via the IMA using three coils (Interlock 2 mm/6 cm × 3, Boston Scientific, MA, USA). The opacification of the aneurysm disappeared on retrograde and antegrade angiography, confirming complete cessation of blood flow into the aneurysm (Fig. 2e, f). The post-procedural course was uneventful. Anemia improvement and absence of bowel ischemia were observed. On follow-up CTA performed 3 months later, the aneurysm was not opacified and the hematoma had decreased in size (Fig. 3).

## Discussion

Visceral arterial aneurysms are rare, with a prevalence of 0.1 to 2% (Tulsyan et al. 2007). SRA aneurysms are extremely rare and have been documented in only 14 case reports, including the present report (Table 1). The most common causes of SRA aneurysms were trauma and NF1 in three cases. All NF1-related SRA aneurysms were observed in the arterial trunk and had ruptured at the time of diagnosis. The vascular fragility of middle to large arteries in NF1 can be related to the location of these aneurysms. The lesions were found to be located away from the rectal mucosa; thus, no patient complained of melena, and CTA was required for diagnosis.

**Table 1:** Characteristics of the reported cases with superior rectal artery aneurysms

First author, year	Age	Sex (M/F)	Melena (Y/N)	Trunk or branch	Ruptured (Y/N)	Cause	Treatment
Pond et al. 1977	80	M	Y	Branch	Y	Unknown	Surgery
Baig et al. 2003	38	M	Y	Branch	Y	Unknown	TAE with coils
Iqbal et al. 2011	23	M	Y	Unknown	Y	Trauma	TAE with NBCA
Janmohamed et al. 2011	63	F	Y	Branch	Y	Diverticulitis	TAE with coils
Kim et al. 2012	83	M	Y	Branch	Y	Dieulafoy lesion	TAE with NBCA after failed with GS
Zakeri and Cheah 2012	26	M	Y	Branch	Y	Trauma (iatrogenic)	TAE with NBCA
Liu et al. 2013	57	M	N	Trunk	N	Arteriosclerosis	Surgery
Makino et al. 2013	39	M	N	Trunk	Y	Neurofibromatosis type 1	TAE with coils
Yow et al. 2015	55	F	N	Trunk	Y	Neurofibromatosis type 1	Surgery after failed TAE with coils
Li et al. 2018	65	M	Y	Branch	Y	Bevacizumab therapy	TAE with coils
Curfman et al. 2020	79	M	Y	Branch	Y	Trauma	TAE with coils
Marusca et al. 2020	45	M	Y	Branch	Y	Unknown	TAE with coils
Nguyen et al. 2020	79	M	Y	Branch	Y	Unknown	TAE with coils
Present case	52	F	N	Trunk	Y	Neurofibromatosis type 1	TAE with coils

*TAE* Transcatheter arterial embolization, *NBCA* N-butyl cyanoacrylate, *GS* Gelatin sponge

TAE is the first choice of treatment for visceral arterial aneurysms owing to shorter hospitalization time and fewer cardiovascular complications (Barrionuevo et al. 2019);

however, in cases that require distal revascularization or bypass, surgery is the only choice. Although massive bleeding during surgery and perioperative mortality due to vascular fragility have been reported in NF1 (Chew et al. 2001), TAE has been reported to provide favorable outcomes with fewer major complications (15%), local recurrence (9.1%), and persistence of symptoms (4.5%) (Bargiela et al. 2018).

In two previous case reports of ruptured SRA trunk aneurysms related to NF1, TAE succeeded in one of the two cases (Makino et al. 2013), but failed in another case (Yow et al. 2015), requiring surgical aneurysmal exclusion for re-bleeding. In the latter case, the SRA could not be catheterized with a microcatheter, and only proximal embolization of the IMA was performed using coils. The authors considered that retrograde reperfusion of the aneurysm sac via patent collaterals could result in recurrent hemorrhage. To prevent this, the aneurysm should be isolated by embolizing the SRA trunk proximal and distal to it. In our case, antegrade distal embolization failed because the aneurysmal cavity was so large that the microcatheter or guidewire could not be directed to the orifice of the distal SRA trunk. Therefore, the distal SRA was embolized retrogradely via MRA-SRA anastomosis from the right internal iliac artery (IIA). To our knowledge, this is the first case of successful coil embolization of an IMA branch through the IIA. The double coaxial microcatheter system enabled the passage of a narrow and tortuous anastomosis. The glue consisting of n-butyl cyanoacrylate and iodized oil can be an alternative embolic agent for the treatment of ruptured aneurysms or pseudoaneurysms when distal catheterization is difficult. It was used in 3 of the 14 reported cases of SRA aneurysms (Table 1) because these patients’ conditions were unstable due to massive bleeding. The glue embolization has the advantage of quick hemostasis. However, it should be noted that embolizing a proximal aneurysm with the glue as in our case increases the risk of post-procedural bowel ischemia.

## Conclusion

SRA trunk aneurysms are rare, but frequently associated with NF1. CTA is recommended for an early diagnosis because the aneurysm can rupture and cause hemorrhagic shock without causing melena. Isolation of aneurysms using embolization coils is necessary to prevent recurrent hemorrhage. Antegrade distal embolization beyond the aneurysm is sometimes difficult to achieve. In such instances, a retrograde approach employing MRA-SRA anastomosis can be used.

## Data Availability

The datasets used during the current study are available from the corresponding author on reasonable request.

## Abbreviations

CTA: Computed tomography angiography
IIA: Internal iliac artery
IMA: Inferior mesenteric artery
MRA: Middle rectal artery
NF1: Neurofibromatosis type 1
SRA: Superior rectal artery
TAE: Transcatheter arterial embolization
